# Affective Responses to Both Climbing and Nordic Walking Exercise Are Associated With Intermediate-Term Increases in Physical Activity in Patients With Anxiety and Posttraumatic Stress Disorder - A Randomized Longitudinal Controlled Clinical Pilot Trial

**DOI:** 10.3389/fpsyt.2022.856730

**Published:** 2022-06-09

**Authors:** Carina S. Bichler, Martin Niedermeier, Katharina Hüfner, Mátyás Gálffy, Barbara Sperner-Unterweger, Martin Kopp

**Affiliations:** ^1^Department of Sport Science, University of Innsbruck, Innsbruck, Austria; ^2^Department of Psychiatry, Psychotherapy, Psychosomatics and Medical Psychology, University Hospital for Psychiatry II, Innsbruck, Austria

**Keywords:** affective valence, climbing therapy, exercise intervention, psychiatric disorder, anxiety, posttraumatic stress disorder

## Abstract

**Background:**

Exercise programs have shown anxiolytic effects in psychiatric patients. Adherence to exercise programs and subsequent long-term lifestyle change is influenced by acute affective responses of the exercise programs. This research aimed to assess acute affective responses of two different exercise modalities compared to a non-exercise control program and its effects on persisting physical activity behavior change.

**Methods:**

Sixty-six outpatients diagnosed with an anxiety disorder or posttraumatic stress disorder were randomly allocated to one of three groups in a randomized longitudinal controlled clinical pilot trial: climbing (*n* = 26), nordic walking (*n* = 19), social contact control (*n* = 21). Affective responses were assessed pre, during, and post activity. General physical activity behavior was recorded prior to participation in the program, post program, and at follow-ups three and six months after the program.

**Results:**

Multilevel modeling analyzes of 1,066 individual data points revealed increases in affective valence in the exercise sessions compared to the social contact sessions. State anxiety decreased in the climbing group compared to the social contact group. Physical activity behavior was increased immediately following the program as well as at six months follow-up in both exercise groups. A larger increase in affective valence during and after the sessions was associated with higher physical activity post program.

**Conclusions:**

Climbing and conventional nordic walking exercise sessions revealed positive affective changes in outpatients indicating therapeutic potential of both modalities for acute emotion regulation. In accordance with theoretical models of human behavior change, it was judged that the experience of a more pleasant affective state following the exercise sessions induced more persisting effects on physical activity behavior after the exercise programs.

**Trial Registration:**

https://www.clinicaltrials.gov/ct2/show/NCT03758599, identifier: NCT03758599.

## Introduction

Physical activity is valued for its positive effects on physical and psychological health (1, 2). The World Health Organization (WHO) recommends a minimum amount of 150 minutes at least moderate intense physical activity per week (1). About 28% of the world population and 42% of the high-income Western countries do not meet these recommendations (3). A closer look at the people who are insufficiently physically active reveals particularly vulnerable groups: One of these groups are people who suffer from mental health problems, although it is well known that this population gains physical and psychological health benefits from physical activity (2). Given the reduced level of physical activity in people with mental disorders, interventions to increase physical activity behavior are urgently needed.

Studies on exercise programs in the treatment of psychiatric disorders focus predominantly on the effects on symptom severity: For individuals with anxiety disorders, anxiolytic effects can be achieved through exercise programs (4). Individuals with trauma-related disorders such as PTSD can benefit from exercise programs because attendant symptoms such as depression, anxiety, or low sleep quality were reduced (5–7). However, the effect of physical activity interventions in the treatment of depression is not uniformly clarified. In 2012, a major nationally funded RCT in the UK for example failed to find such desired effects (8). Nevertheless, the majority of exercise programs that have been studied in individuals with psychiatric conditions, specifically with anxiety disorders or PTSD, contain aerobic exercise, resistance training, or yoga (4, 5, 9, 10). While aerobic exercise could not be uniformly recommended as an effective treatment a few years ago (9), more recent analyses showed symptom reduction due to aerobic exercise (11). A systematic review and meta-analysis found both aerobic and resistance exercise as well as yoga to be beneficial (10). Some studies implemented alternative programs such as climbing exercise for depressive patients (12–15). A manualized bouldering psychotherapy program showed reduced depressive symptoms compared to a home-based exercise program (12). The practice of climbing exercise showed positive physical benefits (13), increased self-efficacy (14, 15), and decreased depressive and anxiety symptoms (12).

While the positive short-term and long-term effects of exercise on mental health are widely researched and discussed, one of the major challenges is to design interventions which induce persistent behavior change with longer lasting effects within an evidence-based comprehensive framework (16). Affective responses to single bouts of exercise are associated with motivational processes connected to physical activity behavior (17, 18). Positive affective responses during exercise bouts can increase long-term participation and engagement in physical activity in persons without a mental health disorder (19). In persons with psychiatric disorders, some findings on positive affective responses after single physical activity bouts have been reported. Bouts of aerobic exercise such as walking showed increased positive valence after the activity in individuals with depression (20).

Recently, a controlled, randomized study comparing occupational therapy, swimming, and climbing was carried out in children and adolescents with mental and behavioral disorders: Results showed that climbing has a positive effect on immediate affective responses during the exercise bout compared to both swimming and occupational therapy (21). In a non-randomized controlled trial in depressive inpatients, a single session of rock climbing showed increased positive affect and coping emotions as well as decreased negative affect and depressiveness compared to relaxation (22). These potential differences in affective responses as well as specific exercise preferences should be considered when choosing an exercise modality for individuals with psychiatric disorder (23).

However, study designs in which different exercise program effects are compared are rare, although the importance of this comparison is increasingly recognized (12, 24, 25). To the best of our knowledge, no study is available that assessed immediate affective responses of different exercise programs in patients with anxiety disorders or PTSD. Also, longitudinal exercise studies in individuals with anxiety or PTSD are missing.

Therefore, the primary aim of the present project was to compare affective responses between different exercise programs based on multiple sessions of the programs. For this purpose, an already established program (nordic walking exercise) was compared to a relatively new exercise program (climbing exercise) in groups of individuals with anxiety disorder or PTSD. Exercise programs were controlled for effects of social contact by implementing a social contact control program with the same amount of social contact, but without exercise. The secondary aim was to compare the effects of the two exercise programs on future physical activity behavior and to analyze the role of affective valence during the sessions of the programs on future physical activity behavior.

## Materials and Methods

### Study Design and Procedures

The overarching aim of the research project was to analyze the effects of two exercise programs (climbing and nordic walking) in comparison to a social contact control group on various health-related outcomes, including symptom severity, in patients with anxiety disorder or posttraumatic stress disorder. In the present study, we focus on the affective responses and their role in future physical activity behavior as one of the health-related outcomes. Participation in the groups was offered as an add-on to the regular clinical outpatient care program in a low-threshold, interdisciplinary network in the area of Innsbruck, Tyrol.

Participants were screened and enrolled by clinical professionals (e.g., Psychologists, Psychiatrists) in the Department of Psychiatry, Psychotherapy and Psychosomatics at the Medical University of Innsbruck. When assessed as eligible for the study, outpatients were invited to an informative meeting and randomly allocated to one of three groups by use of a computer-generated, blockwise cluster-randomization scheme with an allocation ratio of 1:1:1. Block sizes were a minimum of three and a maximum of eight. Whether participants had a diagnosis of AD vs. PTSD was not considered in randomization. This procedure was of an organizational nature, so that the participants were offered the earliest possible start of the study program to reduce drop out.

**Figure 1:**
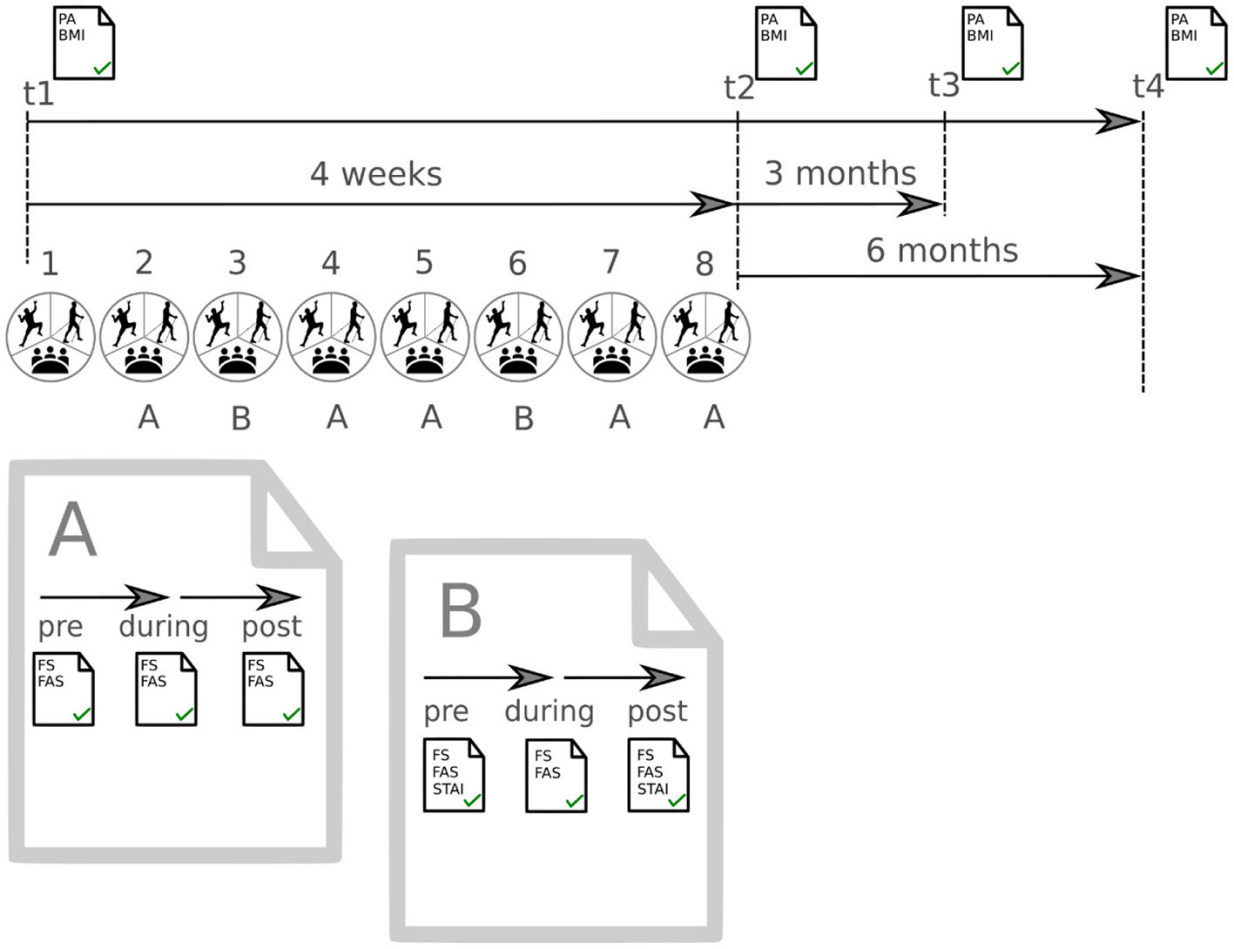
Schedule of the complete project. Measurement of physical activity (PA) and Body Mass Index (BMI) at 4 timepoints (t1–t4). Procedure of measurement for affective responses from session 2–8. Set A Feeling Scale (FS) and Felt Arousal Scale (FAS) were recorded in all sessions. Set B was recorded in sessions 3 and 6 and additionally included the State-Trait-Anxiety Inventory (STAI).

Participants received study information and timetables for their group attendances by administrative staff of the Department. The program started within the following two weeks and the period ended after eight group sessions within four weeks. Follow-up measures were conducted immediately after, three, and six months after the program ended (Figure 1).

In the first session, focus was set on the practical implementation. Participants were introduced to the program to be able to familiarize with the situation. The intervention was performed and personal assistance was provided as required.

Starting with session two, affective responses (affective valence and perceived activation) were assessed with standardized questionnaires at three points in time at each session: pre (beginning of the session), during (after 45 minutes), and post (immediately after) session (Figure 1). In sessions three and six, state anxiety was additionally assessed with a standardized questionnaire, at both pre and post session. State anxiety was assessed only two times to reduce the questionnaire load for the participants. A description of the questionnaires is given in Outcomes. Sociodemographic (age, sex, psychiatric diagnosis) and health-related characteristics (e.g., self-reported physical activity, measured body mass index) were conducted pre program (t1), post program (t2), at follow-up 3 months post program (t3), and at follow-up 6 months post program (t4).

### Participants

The participants were recruited by local treatment staff.

Inclusion criteria were: (a) outpatients with a primary ICD-10 diagnosis of any anxiety disorder or posttraumatic stress disorder (PTSD) (F 40, F 41, F 43.1; ICD-10; World Health Organization), (b) aged between 18 and 65 years, and (c) giving written informed consent.

Exclusion criteria were: (a) patients with acute psychosis or suicidal behavior, (b) medical contraindication to exercise (assessed by a clinician), (c) somatic comorbidity with contraindication to moderate physical activity (e.g., high risk of cardiac events) (judged by patients primary care physician), (d) cognitive deficits (unsuitable to complete the required questionnaires, diagnosed by the referring psychiatrist, psychologist or psychotherapist), and (e) problems with German language (diagnosed by the referring psychiatrist, psychologist or psychotherapist).

No a priori power analysis is available for the primary outcome affective responses. Since the present study is part of a research project to assess different health-related outcome variables, only one power analysis based on symptom severity was conducted for the research project, an outcome not considered in this study. However, the sample size of the present study is in the range of previously published studies, e.g., (12, 26, 27).

### Programs

All programs were led by two professionals, including clinical health psychologists, sport and exercise psychologists, sport scientists, training therapists, and physicians. All programs consisted of eight 90-minutes sessions spread over four weeks (two sessions per week). Both exercise programs were performed with comparable intensity. Participants in both exercise programs were instructed to perform the sessions with moderate intensity (being able to talk but not sing during the activity).

#### Climbing Exercise Group (Climbing Group)

At the beginning of each session, a standardized body-centered warm-up of ten minutes identical with the nordic walking group took place. The group sessions were held in an outdoor climbing gym when weather and temperature conditions allowed. In bad weather conditions, the indoor climbing gym was used. The general warm-up was followed by a climbing specific warm-up, which consisted of bouldering (i.e., climbing horizontally without the use of a rope near the floor) for around 25 minutes. After warm-ups, the actual rope climbing sessions lasted around 45 minutes. During the first five sessions of rope climbing, only the therapist provided rope security. Safety and belaying of partners were trained throughout those sessions. Depending on participants' knowledge and skills, they were allowed to belay each other; however, always guided by a therapist. As in Gallotta et al. (13), climbing sessions contained several sport-specific skills-development training sessions to familiarize the participants with gear and rope management, to learn footwork and route finding, and to locate good belay spots and resting positions while climbing. Every climbing session ended with a short cool-down session of five minutes (i.e., stretching) identical to the nordic walking group.

#### Nordic Walking Exercise Group (Nordic Walking Group)

The nordic walking group started with a ten-minute body-centered warm-up identical to the climbing group, followed by a five-minute nordic walking specific warm-up. Afterwards, the group walked with a moderate pace outdoors on varying paths for 70 minutes using the nordic walking technique. Specially designed poles were used in this technique to push against the ground with each stride, activating the upper body while walking. The session ended with a five-minute cool-down session identical to the climbing group.

#### Social Contact Control Group (Social Contact Group)

Social interaction is discussed as one of the anxiolytic mechanisms of exercise programs in group settings (28). The social contact control group was included to be able to compare potential effects of exercise in an interactive group setting to effects of an interactive group setting without exercise. Participants allocated to the social contact group received brief background information and then watched a movie in the indoor group sessions. Movies included four animated and feature films on anxiety related topics. One full movie was presented within two sessions, followed by a guided group dialog. The group dialog lasted approximately 30 minutes, depending on movie length and focus on a communicative exchange regarding the movie and anxiety related topics, led by the therapist. However, the participants received the same amount of social interaction and support as the exercise groups.

### Outcomes

#### Primary Outcomes

The dimension of affective valence was assesse by the Feeling Scale [FS; (29)]. This single-item rating scale ranges from “+5” (very good) to “−5” (very bad), with anchors at “0” (neutral) and at all odd integers. Convergent validity information for the FS has been provided (30–32). The FS has been used previously in exercise studies within a clinical population, e.g., (21, 25, 33–35).

The dimension of perceived activation was assessed by the Felt Arousal Scale [FAS; (36)]. This single-item rating scale ranges from “1” (low arousal) to “6” (high arousal). The FAS has been used in previous studies on physical activity, demonstrating convergent validity with other measures of perceived activation (32, 36).

The State-Trait-Anxiety Inventory [STAI; (37)] is a 20-item rating scale for each state anxiety (STAI-S), and trait anxiety (STAI-T), ranging from “1” (not at all/almost never) to “4” (very much so/almost always). In this project, only the STAI-S was used. State anxiety is defined as a mood characterized by tension, anxiety, nervousness, restlessness, and fear of future events and is accompanied by increased activity of the autonomic nervous system (38, 39). Unlike other measures of anxiety, the STAI operationalizes “anxiety” close to the body, so that the connectivity of arousal data is facilitated. The participants were asked to rate the extent of anxiety of the present moment (right now). The STAI has been previously used in exercise studies [e.g., (40)].

#### Secondary Outcomes

Physical activity was assessed in a practitioner interview that took place four times during study participation. During a pilot phase prior to the present study, patients provided feedback and were observed to have difficulty completing the International Physical Activity Questionnaire [IPAQ; (41)]. Therefore, the procedure was adapted, and standardized interview questions were asked for the type of physical activity, and secondly for the number of hours of this activity in the previous seven days. Following the IPAQ, the interviewer assisted in the assessment of activity and distinguished between a) vigorous, b) moderate, and c) light intense physical activity such as walking. As an indicator of physical activity, the intensity hours were calculated by multiplying physical activity units of at least ten minutes with an intensity weight and adding all intensity hours per week. According to the guidelines of the IPAQ (41), the sum of the specified hours of the respective physical activity was formed as follows:

“*hours of light intense* physical activity*/walking” x 0.75* + *hours of “moderate intense* physical activity” + *hours of “vigorous intense* physical activity”* x 2*.

### Statistical Analysis

The data on affective responses were collected across several sessions and were nested within patients. Since there is no data on comparing several program sessions available, the project included all compiled data. Ordinary methods of analyses of variances might produce biased inferential statistics since the inter-correlation of repeated observations in the same patients over time cannot be accounted for (42). Therefore, multilevel modeling was conducted for the primary research question using the GAMLj module (43) in jamovi v. 1.2 (44). Data of all patients attending at least two sessions of the allocated program were analyzed. The variables patient and session were modeled as cluster variables to account for the hierarchical data structure. The factors time (two or three levels), group (three levels), and the time-by-group interaction were included as fixed effects. The time-by-group interaction analysis was the primary analysis of interest. Pre-planned simple contrasts were conducted for the factor time using the time point “pre” as the reference category and for the factor group using “climbing group” as the reference category. Random effects were first defined for patient and session allowing the intercepts to vary across patients and sessions. Whenever the variation across patients and/or sessions was too small (indicated by the likelihood ratio test on random effects), the random effect was excluded. Three outcome variables for affect were analyzed: affective valence, perceived activation (each assessed three times in seven sessions), and state anxiety (each assessed two times in two sessions) (see Figure 1).

Cohens d (45) was calculated as an effect size for pairwise comparisons subtracting mean change over time of the nordic walking group/social contact group from mean change over time in the climbing group divided by the pooled standard deviation. Consequently, positive effect sizes indicate a larger increase in the climbing group compared to the other groups.

For the secondary research question, the differences in physical activity between groups were analyzed using SPSS version 26 (IBM, New York, USA) on a per protocol basis. In a series of analyses of covariance with physical activity at t1 (pre program) as a covariate, group differences in physical activity at t2 (post program), at t3 (follow-up three months post program), and at t4 (follow-up six months post program) were analyzed. Pre-planned simple contrasts with “climbing group” as the reference category were conducted. Following the analyses on group differences, generalized estimating equations (GEE) were calculated to assess the role of affective valence in future physical activity. A linear relationship was chosen in accordance to previous literature (19) and a working correlation matrix with exchangeable structures was used for all GEE models. Like multilevel modeling, GEE allows to account for the hierarchical data structure by using patient and session as cluster variables. In total, six separate GEE models were calculated to model the three outcome variables “physical activity” at t2, t3, and t4. The outcome variables were modeled by the factors physical activity at t1 to control for differences in physical activity at baseline and by affective valence according to the Feeling Scale. One GEE model each was calculated for affective valence during and post session to be able to compare regression coefficients of affective valence during session and post session. To control for daily fluctuations, the changes in affective valence (during—pre session and post—pre session) were used in the GEE models. Accordingly, higher changes in affective valence indicate more positive changes in affect. Other potential confounders (e.g., sex, age, or body mass index) were believed to be more appropriately reflected in physical activity at t1 and were therefore not included in the model. Quasi-information criterion (QIC) was given for the GEE model with a lower QIC value indicating better model fit (46).

*P*-values < 0.05 were considered as significant. Unless otherwise stated, data are presented as mean ± SD.

## Results

**Figure 2:**
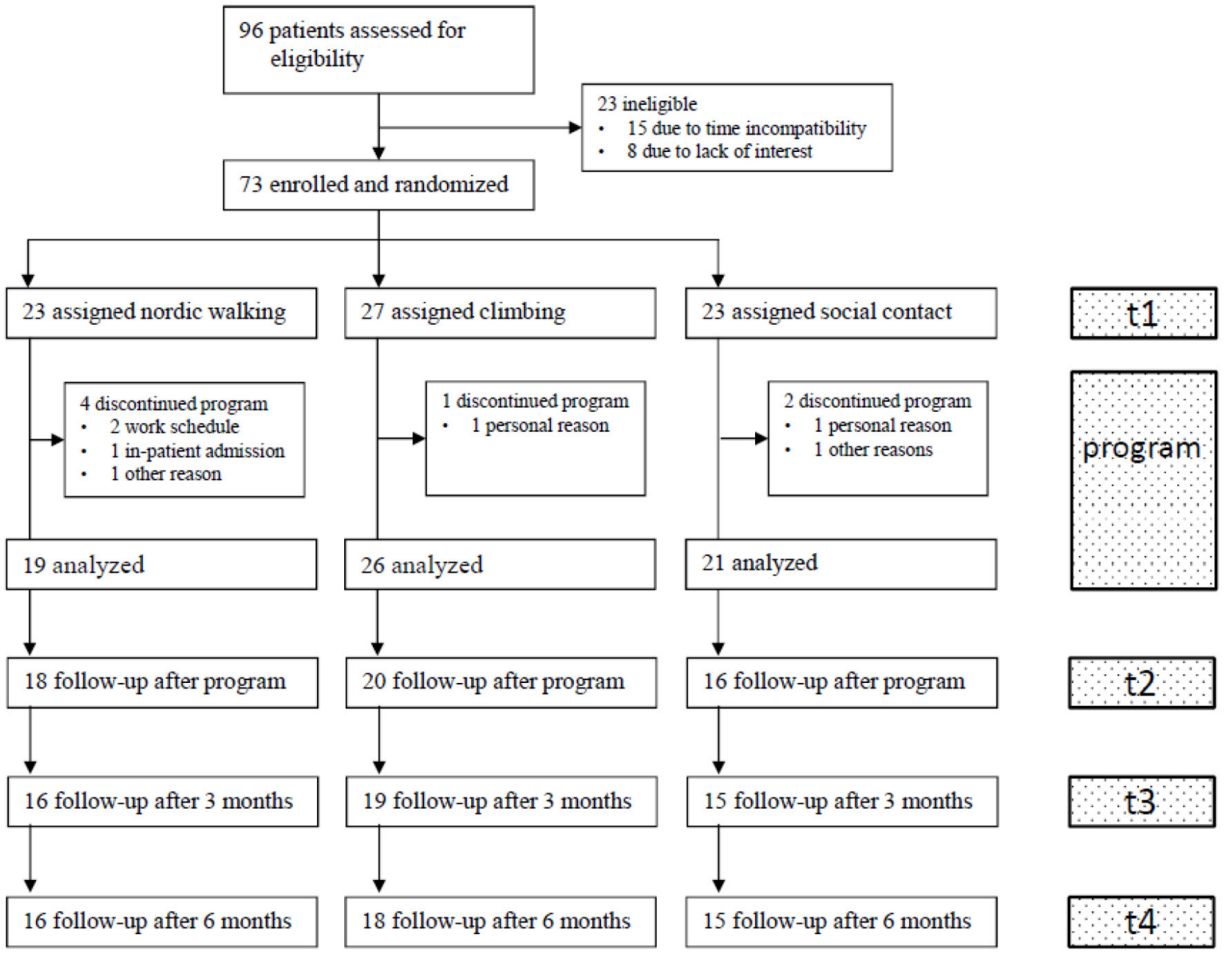
Participant flow.

Between Oct 16, 2017, and Dec 18, 2019, 96 patients followed the invitation for the information meeting and 23 declined to participate due to time incompatibility (*n* = 15) or lack of interest (*n* = 8). Seventy-three patients were randomized, and seven patients dropped out after the first session [one for the climbing group due to personal reasons; four for the nordic walking group due to time incompatibility (*n* = 2), inpatient admission (*n* = 1), unclear reason (*n* = 1); and two for the social contact control group due to personal reasons (*n* = 1) and unclear reason (*n* = 1)]. The total sample for the analysis of affective responses consisted of 66 patients (mean age 44.2 ± 13.2 years). Twenty-six patients participated in the climbing group, 19 in the nordic walking group, and 21 in the social contact group (Figure 2). Follow-up measurements were conducted between Nov 11, 2017, and Aug 11, 2020. Table 1 shows sociodemographic and health-related characteristics in each of the three groups. No harmful event was observed in any of the patient groups.

**Table 1:** Sociodemographic and health-related characteristics of participants.

**Variable**	**Climbing group**	**Nordic walking group**	**Social contact group**
Number (%)	26 (39%)	19 (29%)	21 (32%)
Age in years: M ± SD	44.6 ± 13.7	44.7 ± 13.1	43.3 ± 13.3
Sex *absolute numbers* (%)	18 (69%) female; 8 (31%) male	16 (84%) female; 3 (16%) male	16 (76%) female; 5 (24%) male
***Diagnosis***			
Anxiety disorder *absolute numbers* (%)	20 (77%)	15 (79%)	12 (57%)
PTSD *absolute numbers* (%)	5 (19%)	3 (16%)	7 (33%)
Anxiety Disorder and PTSD *absolute numbers* (%)	1 (4%)	1 (5%)	2 (10%)
***Body mass index***			
t1: M ± SD	25.0 ± 4.4	26.2 ± 6.6	27.4 ± 4.1
t2: M ± SD	24.6 ± 4.6	26.4 ± 6.2	27.9 ± 4.2
t3: M ± SD	24.5 ± 4.6	26.0 ± 6.1	28.1 ± 4.6
t4: M ± SD	24.5 ± 4.2	25.9 ± 6.0	28.1 ± 4.9

*M, mean; SD, standard deviation; PTSD, posttraumatic stress disorder; t1, pre program; t2, post program; t3, follow-up 3 months post program; t4, follow-up 6 months post program*.

### Affective Valence and Perceived Activation

For affective valence, 1,066 observations from 66 different patients were available (climbing group: 411, nordic walking group: 345, social contact group: 310). For perceived activation, 1,065 observations from 66 different patients were available (climbing group: 410, nordic walking group: 345, social contact group: 310). The intercepts for affective valence varied significantly across patients, variance = 2.4, *p* < 0.001, and across sessions, variance = 0.05, *p* = 0.004. The intercepts for perceived activation varied significantly across patients, variance = 0.7, *p* < 0.001, but not across sessions, variance < 0.01, *p* < 1.000.

**Figure 3:**
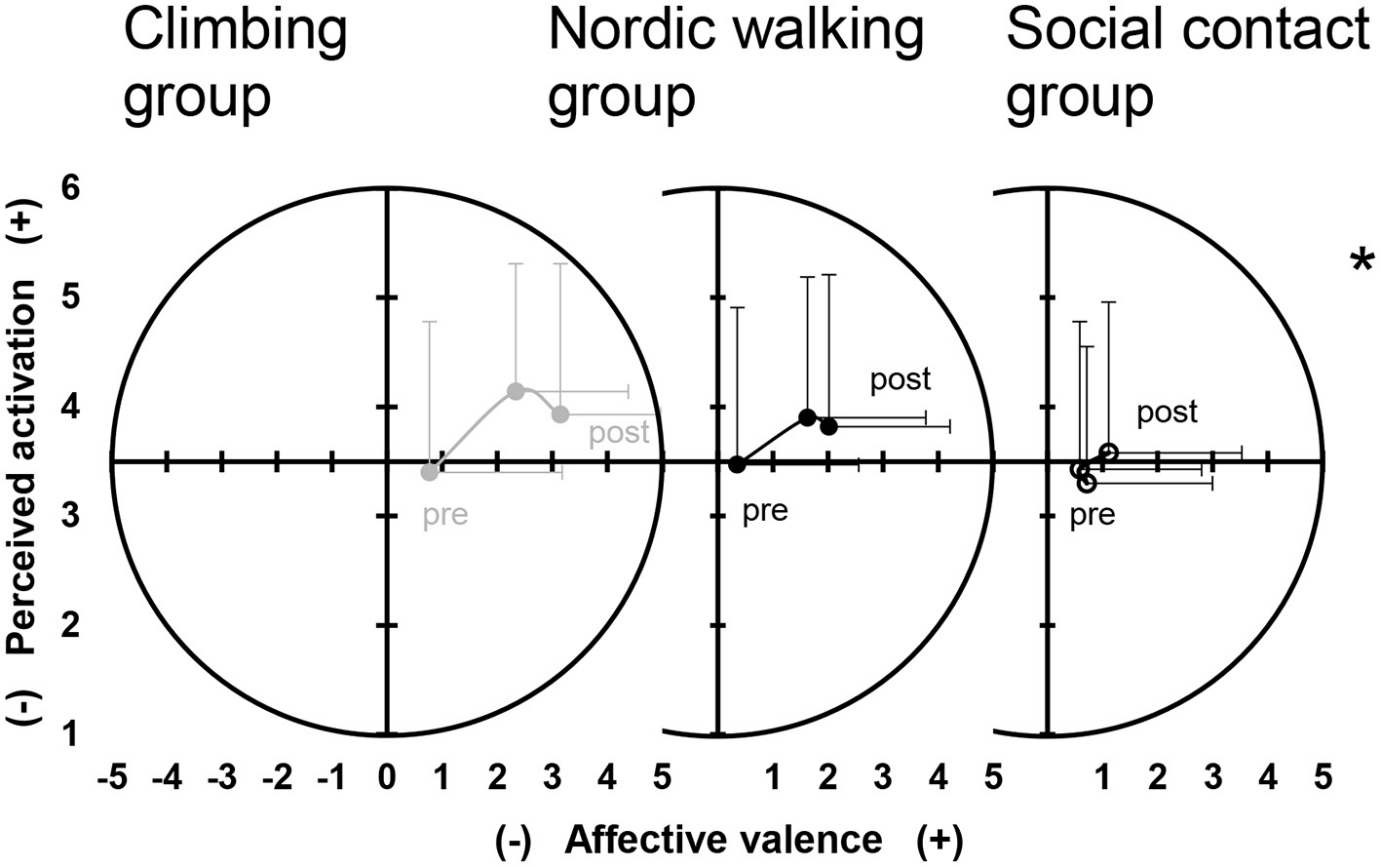
Dimensional affective responses, affective valence, and perceived activation over time separated by climbing group (left panel, filled gray circles), nordic walking group (middle panel, filled black circles), and social contact group (right panel, empty black circles). Circles represent mean values and error bars indicate standard deviations. *Indicates a significant time-by-group interaction both for affective valence and perceived activation.

A significant time-by-group interaction emerged for affective valence, *F*(4, 988.7) = 14.83, *p* < 0.001 (Figure 3). Simple contrasts showed a significantly larger pre vs. during increase in affective valence in the climbing group compared to the social contact group, *p* < 0.001, *d* = 0.77, but no significantly different pre vs. during change compared to the nordic walking group, *p* = 0.292, *d* = 0.13. A significantly larger pre-post increase in affective valence was found in the climbing group compared to both the social contact group, *p* < 0.001, *d* = 0.52, and to the nordic walking group, *p* = 0.010, *d* = 0.33. Significant main effects on affective valence were found for the factors time, *F*_(2, 988.7)_ = 81.30, *p* < 0.001, and group, *F*_(2, 63.0)_ = 6.28, *p* = 0.003.

Perceived activation showed a significant time-by-group interaction, *F*_(4, 994.0)_ = 2.53, *p* < 0.001 (Figure 3). No significantly different changes between groups were found for the pre-during comparison, *p* >0.094, *d* < 0.24. A significantly larger pre-post increase in perceived activation emerged for the climbing group compared to the social contact group, *p* = 0.002, *d* = 0.46, but not compared to the nordic walking group, *p* = 0.230, *d* = 0.34. Significant main effects on perceived activation were found for the factor time, *F*_(2, 994.0)_ = 18.64, *p* < 0.001, but not for the factor group, *F*_(2, 63.3)_ = 0.49, *p* = 0.617.

### State Anxiety

For state anxiety, 188 observations from 60 different participants were available (climbing group: 65, nordic walking group: 66, social contact group: 57). The intercepts for state anxiety varied significantly across participants, variance 108.6, *p* < 0.001, but not between sessions, variance < 0.01, p < 1.000.

**Figure 4:**
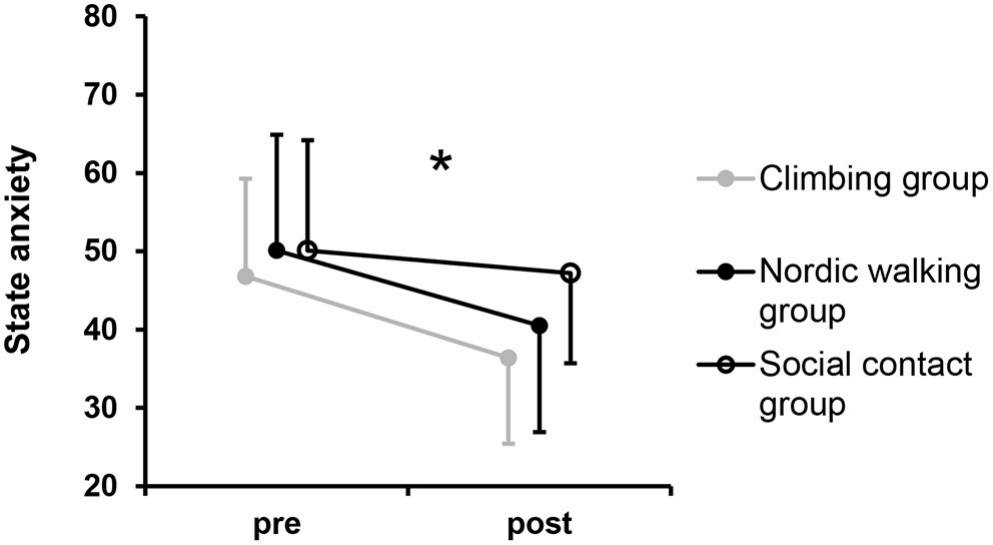
State anxiety over time separated by groups. Circles represent mean values and error bars indicate standard deviations. *Indicates a significant time-by-group interaction.

A significant time-by-group interaction emerged for state anxiety, *F*_(2, 125.6_) = 4.14, *p* < 0.018 (Figure 4). Simple contrasts showed a significantly larger decrease in state anxiety in the climbing group compared to the social contact group, *p* = 0.010, *d* = −0.55, but no significantly different change compared to the nordic walking group, *p* = 0.810, *d* = −0.07. Significant main effects on state anxiety were found for the factor time, *F*_(1, 125.6)_ = 44.10, *p* < 0.001, but not for factor group, *F*_(2, 56.9)_ = 2.17, *p* = 0.123.

### Group Differences in Physical Activity

**Figure 5:**
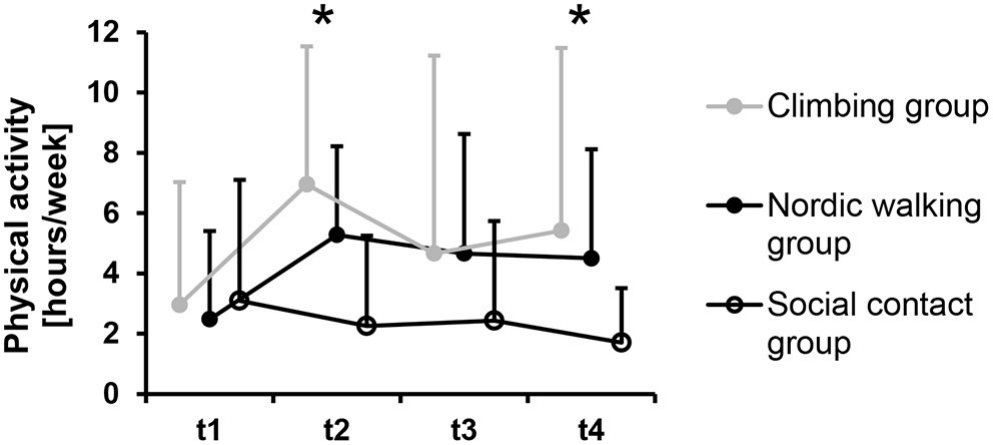
Physical activity pre program (t1), post program (t2, *n* = 54), at follow-up 3 months post program (t3, *n* = 50), and at follow-up 6 months post program (t4, *n* = 49) separated by groups. Circles represent mean values and error bars indicate standard deviations. *Indicates a significant group difference in the analysis adjusted by physical activity pre program.

When adjusted by physical activity at t1, significant group differences in physical activity were found at t2, *F*_(2, 50)_ = 18.98, *p* < 0.001, and at t4, *F*_(2, 45)_ = 3.89, *p* = 0.028 (Figure 5). Simple contrasts revealed a significantly higher physical activity of the climbing group compared to the social contact group, t2: *p* < 0.001, *d* = 1.21, t4: *p* = 0.016, *d* = 0.91, but not compared to the nordic walking group, t2: *p* = 0.404, *d* = 0.32, t4: *p* = 0.920, *d* = 0.10. No significant difference was found at t3, *F*_(2, 46)_ = 1.97, *p* = 0.151, *d* < 0.51.

### Physical Activity and Affective Valence

Generalized estimating equations showed the best model for the prediction of physical activity post program at t2 (Table 2). Physical activity pre program showed significant associations with physical activity post program at all time points. Higher physical activity pre program was associated with higher physical activity post program. Changes in affective valence were positively associated with physical activity only post program at t2. A more positive affective state during, *B* = 0.41, and post sessions, *B* = 0.37, was associated with higher physical activity at t2. No significant associations between changes in affect and physical activity were found for t3 and t4, *p* > 0.280. The models using the change in affective valence from pre-during session and pre-post session were widely similar.

**Table 2:** Generalized estimating equations with the outcome physical activity post program (t2), at follow-up 3 months post program (t3), and at follow-up 6 months post program (t4) separately for pre-during and pre-post changes in affective valence.

	**Variable**	**During—pre**				**Post—pre**			
		***B***	***SE B***	***p***	***QIC***	***B***	***SE B***	***p***	***QIC***
t2	Physical activity pre program	0.86	0.09	<0.001*		0.86	0.09	<0.001*	
	Change in affective valence	0.41	0.09	<0.001*	1,957	0.37	0.08	<0.001*	1,956
t3	Physical activity pre program	0.99	0.22	<0.001*		1.00	0.22	<0.001*	
	Change in affective valence	0.08	0.18	0.660	4,038	−0.01	0.17	0.960	4,046
t4	Physical activity pre program	0.73	0.25	0.003*		0.73	0.24	0.003*	
	Change in affective valence	0.20	0.18	0.281	4,267	0.19	0.18	0.279	4,260

B, unstandardized regression coefficient; SE B, standard error of B; QIC, Quasi-information criterion; *Indicates significant coefficients.

## Discussion

The main findings of the present study are

(1) Both types of exercise sessions resulted in more pleasurable and higher activated affective states as well as a short-term anxiolytic effect in individuals with anxiety or posttraumatic stress disorder, compared to social contact sessions without exercise.
(2) Higher positive affective states during and after the exercise sessions were associated with intermediate term increases in physical activity.
(3) The modality of exercise appears to be secondary for affective states, although small-sized beneficial effects were found in affective valence for climbing sessions compared to nordic walking sessions.
(4) The increase in physical activity persisted six months following completion of the clinical pilot trial.

### More Pleasant, Higher Activated State During and After Exercise Sessions

The finding of a more pleasant, higher activated state after exercise sessions is consistent with findings in existing literature on single exercise bouts in psychiatric patients, e.g., (20, 21, 25, 33). The positive changes in affective states (especially in pleasure) are desirable from a motivational perspective for future physical activity behavior (18). The present study extends existing findings of single bouts of exercise on combined information over several sessions. As suggested by Unick et al. (47), findings are more valid if several sessions are taken into account.

In the present study, a more pleasant, higher activated state during and after exercise was found in comparison to social contact sessions. Currently, the effect of social contact may be underestimated and only few studies control for these effects, e.g., (21, 25).

However, when the exercise sessions are not compared to a physically inactive control condition, but the two exercise modalities are compared, similar beneficial changes in affect were observed (21, 25). The largest differences between exercise modalities were that climbing exercise resulted in a larger pre-post increase in affective valence and perceived activation compared to the nordic walking group. Reasons for the more pleasant state after climbing sessions may result from psychological, social, and physiological parameters. The relatively new modality of climbing exercise in this setting brings up new challenges of focusing attention and requiring facing and overcoming feelings of anxiety. Focus on the present moment interrupts rumination and enables the building of trust in one's own abilities (self-efficacy) and trust in others (48). Furthermore, climbing is a type of skill-specific training that could involve activation of brain areas associated with executive control that could potentially have positive impact for patients with AD and PTSD (49). Studies in the field of climbing therapy or bouldering psychotherapy also point out these and further positive effects of climbing exercise that go beyond the conventional health benefits of exercise (12, 21). Compared to nordic walking, however, climbing is logistically more complex to conduct.

There is a further influence from indoor and outdoor effects. While in this study climbing was practiced indoors or outdoors, depending on the weather conditions, nordic walking took place outdoors all the time. The social contact group took place indoors under all weather conditions. In studies with depressive in-patients it could be shown that the same physical activity in the outdoors produced better results on the affective state (33, 50, 51). This green exercise effect should be taken into account in the interpretation (52).

When looking at perceived activation, this dimension increased pre-post programs in climbing exercise compared to nordic walking exercise and non-exercise control. From previous studies it is known that even a few minutes of exercise increase perceived activation (53). An increase in perceived activation is desirable for people with symptoms of mental health disorders, e.g., depressive symptoms. However, for a population of individuals with anxiety or PTSD symptoms who often suffer from hyperarousal, an increase in activation is not always considered adjuvant (54). Considering the high affective valence, the slight increase in activation during climbing exercise can be interpreted as positive (53).

### The Potential of Exercise as a Short-Term Anxiolytic Intervention

State anxiety showed a larger decrease with moderate effect size in climbing exercise compared to non-exercise control, but not compared to nordic walking exercise. This finding points out the potential of exercise and confirms earlier research results (55).

It is remarkable that climbing showed a decrease in state anxiety although some components in climbing are associated with increased anxiety (e.g., height, trust in the climbing partner, and perception of an increased heart rate). These findings are in line with previous findings in depressive patients (12).

Interrupting social withdrawal and increasing social interaction is one of the mechanisms seen responsible for the anxiolytic effect of exercise (28). This study explicitly aimed to control for this effect by including a non-exercise control group.

### Physical Activity Behavior After the Programs Including the Role of Affective Valence

Given the low rate of physical activity and the health benefits connected with physical activity (2, 56), it is worth considering the longer-term effects of exercise programs on physical activity behavior.

Findings of the present study showed higher physical activity with a large effect size in the climbing group after the program ended and six months after the program ended in the follow-up, compared to the non-exercise control group. In the social contact control group, the weekly amount of physical activity decreased slightly over the seven-month observation period. We therefore conclude that exercise might trigger consciousness for physical activity in general; a desirable finding from a health behavior perspective (19, 56). Similar to affective responses, the exercise modality seems to be less relevant for longer-term behavior changes toward augmenting physical activity. Previous findings showed positive exercise effects due to different exercise modalities such as resistance training (57) and aerobic exercise (58).

Exploring the role of affective valence in longer term physical activity behavior, the present study showed that a more positive affective state during and post sessions was associated with higher physical activity in the post program period. A change of one unit on the Feeling Scale used to measure affective valence was associated with an increase of 25/22 minutes per week for climbing/nordic walking. This result is similar to an augmentation of 38 minutes that has been reported in the study of Williams et al. (19). However, this was an intermediate term effect as it only applied for the post program period and no comparatively high levels of physical activity in the longer term could be observed for six or twelve months as reported by Williams et al. (19).

Regarding the timing of affective assessment, the present results do not suggest a stronger association of physical activity to affective valence during the exercise compared to affective valence post sessions. For the time points three and six months after program, however, no significant association of physical activity to affective valence was found. The regression coefficients of affective valence during and post exercise were similar suggesting that affective valence post sessions should not be neglected at least for the present population. This finding contradicts Rhodes and Kates (59) who concluded that positive affective responses during the engagement in physical activity is more important in predicting long term physical activity than positive affect after a physical activity session. Therefore, more studies should be conducted in the future that capture the affective responses of different types of exercise before, during, and after the bouts in specific at-risk populations.

### Strengths and Limitations

To the best of our knowledge, this is the first study comparing two exercise modalities and further control for social contact group effects in people with anxiety disorder or PTSD. Another strength of the project was to analyze several sessions instead of a single bout of exercise as well as the long-term follow-up. However, in order to attract participants, it was necessary to make the program suitable for everyday use. This was accompanied by some limitations that have to be considered when interpreting the findings. Firstly, a program period of only eight sessions was limited in scope. Secondly, the project investigators had no control about who agreed or refused to participate in the program. Therefore, the randomization process was weakened because patients may have dropped out because their randomized group did not match their interest (e.g., choice of preferred exercise). Thirdly, the project included outpatients with a psychiatric diagnosis of PTSD or any anxiety disorder. Ideally, the results would be interpreted in a diagnosis-homogeneous manner. Due to the sample size, a differential diagnosis was not considered. Fourthly, the assessment of physical activity was methodically relatively weak. Since reported and actual PA are known to differ, accelerometers would have ideally been used.

## Conclusion

The present findings provide new insights in immediate affective responses to exercise compared to non-exercise programs based on multiple sessions. The more pleasant and higher activated affective state during and after exercise sessions as well as the anxiolytic effect suggest a benefit of exercise beyond social effects of the exercise group settings. Both nordic walking and climbing are considered appropriate exercise modalities for acute emotion regulation in outpatients with anxiety disorder or PTSD. The equipment expenditure for climbing should be critically weighted against small affective advantages of climbing compared to nordic walking when selecting an appropriate exercise modality for patients with anxiety disorder or PTSD. While the affective responses during and following exercise were associated with intermediate term increases in physical activity, the increase in exercise at six months post intervention indicates longer-lasting behavioral changes induced by the exercise programs. The difficulty of achieving sustainable behavioral changes is one of the major challenges in medicine, and this study shows promising results in a hard to reach group with anxiety disorder or PTSD.

## Data Availability Statement

The raw data supporting the conclusions of this article will be made available by the authors, without undue reservation.

## Ethics Statement

The studies involving human participants were reviewed and approved by Ethics Committee of the Medical University of Innsbruck. The patients/participants provided their written informed consent to participate in this study.

## Author Contributions

CB and MK were responsible for the investigation, concept, and design of the study. MG, KH, and BS-U provided the acquisition of data. MN and CB were given visualizations and charge of the methodology. The project administration was given by MG, BS-U, KH, and CB while resources were provided by MK and BS-U. MN sub served data curation and formal analysis that was validated by CB and MK. MK and BS-U supervised the project. CB, MN, and MK wrote the first draft of the manuscript which was reviewed and edited by KH, MG, and BS-U. All authors contributed to manuscript revision, read, and approved the submitted version.

## Funding

This research was supported by the Department of Sport Science, University of Innsbruck and the Department of Psychiatry, Psychotherapy, Psychosomatics and Medical Psychology, University Hospital for Psychiatry II, Innsbruck.

## Conflict of Interest

The authors declare that the research was conducted in the absence of any commercial or financial relationships that could be construed as a potential conflict of interest.

## Publisher's Note

All claims expressed in this article are solely those of the authors and do not necessarily represent those of their affiliated organizations, or those of the publisher, the editors and the reviewers. Any product that may be evaluated in this article, or claim that may be made by its manufacturer, is not guaranteed or endorsed by the publisher.
